# Interaction of Serum- and Glucocorticoid Regulated Kinase 1 (SGK1) with the WW-Domains of Nedd4-2 Is Required for Epithelial Sodium Channel Regulation

**DOI:** 10.1371/journal.pone.0012163

**Published:** 2010-08-13

**Authors:** Dominik Wiemuth, J. Shaun Lott, Kevin Ly, Ying Ke, Paul Teesdale-Spittle, Peter M. Snyder, Fiona J. McDonald

**Affiliations:** 1 Department of Physiology, University of Otago, Dunedin, New Zealand; 2 School of Biological Sciences, University of Auckland, Auckland, New Zealand; 3 School of Biological Sciences, Victoria University of Wellington, Wellington, New Zealand; 4 Departments of Internal Medicine and of Molecular Physiology and Biophysics, Roy J. and Lucille A. Carver College of Medicine, University of Iowa, Iowa City, Iowa, United States of America; Abramson Research Center, United States of America

## Abstract

**Background:**

The epithelial sodium channel (ENaC) is an integral component of the pathway for Na^+^ absorption in epithelial cells. The ubiquitin ligases Nedd4 and Nedd4-2 bind to ENaC and decrease its activity. Conversely, Serum- and Glucocorticoid regulated Kinase-1 (SGK1), a downstream mediator of aldosterone, increases ENaC activity. This effect is at least partly mediated by direct interaction between SGK and Nedd4-2. SGK binds both Nedd4 and Nedd4-2, but it is only able to phosphorylate Nedd4-2. Phosphorylation of Nedd4-2 reduces its ability to bind to ENaC, due to the interaction of phosphorylated Nedd4-2 with 14-3-3 proteins, and hence increases ENaC activity. WW-domains in Nedd4-like proteins bind PY-motifs (PPXY) present in ENaC subunits, and SGK also has a PY-motif.

**Principal Finding:**

Here we show that single or tandem WW-domains of Nedd4 and Nedd4-2 mediate binding to SGK and that different WW-domains of Nedd4 and Nedd4-2 are involved. Our data also show that WW-domains 2 and 3 of Nedd4-2 mediate the interaction with SGK in a cooperative manner, that activated SGK has increased affinity for the WW-domains of Nedd4-2 *in vitro*, and a greater stimulatory effect on ENaC Na^+^ transport compared to wildtype SGK. Further, SGK lacking a PY motif failed to stimulate ENaC activity in the presence of Nedd4-2.

**Conclusions:**

Binding of Nedd4-2 WW-domains to SGK is necessary for SGK-induced ENaC activity.

## Introduction

The epithelial sodium channel is an important component of the body's control of sodium homeostasis and blood pressure [1]. A number of cellular pathways impact on ENaC function: for example, ubiquitination by the Nedd4-family of E3 ubiquitin ligases leads to a decrease in ENaC activity [2], [3], whereas ENaC cleavage by proteases such as furin [4] or prostasin [5] leads to activation of ENaC. The Serum- and Glucocorticoid regulated Kinase isoform 1 (hereafter referred to as SGK) is a positive regulator of ENaC [6]. SGK expression is switched on in response to a number of stimuli and integrates information from several pathways including the insulin, mineralocorticoid and cAMP signalling pathways [7], [8]. Co-expression of SGK with ENaC in *Xenopus* oocytes increases amiloride-sensitive current mediated by ENaC [6], [9].

ENaC activity can be inhibited by three Nedd4-family members: Nedd4, Nedd4-2 and WWP2 [10], [11], [12]. However, the interaction between Nedd4-2 and ENaC appears to be the most important because RNAi studies in mammalian epithelia showed that Nedd4-2 siRNA, but not Nedd4 siRNA, increased amiloride-sensitive Na^+^ current [2], and because a Nedd4-2 knockout mouse develops salt-sensitive hypertension [13]. Nedd4 family members contain three or four WW-domains characterized by two conserved tryptophans (W) which mediate interaction with protein substrates; an enzymatic HECT (homologous to E6-AP C-terminus) domain which catalyzes addition of ubiquitin to target proteins; and a C2 calcium-lipid binding domain is present in some isoforms. WW-domains of Nedd4-like proteins interact with PY-motifs (PPXY) present in the C-terminal domains of the α-, β- and γENaC proteins. Previously we have shown that WW-domain 3 of Nedd4 is critical for the binding and inhibition of ENaC by Nedd4 [14], [15], while others have shown that WW-domain 3, along with WW-domain 4 of Nedd4-2 appear to be critical for ENaC binding [16], [17], [18].

Previously two groups reported that SGK phosphorylated Nedd4-2 on consensus SGK-phosphorylation sites [19], [20], suggesting that the mechanism of SGK-mediated upregulation of ENaC involves the interaction of SGK with Nedd4-2, reviewed in [21]. 14-3-3 proteins bind to phosphorylated Nedd4-2 and are believed to sequester Nedd4-2, reducing its interaction with ENaC [22], resulting in increased ENaC activity [20]. In a feedback mechanism activated Nedd4-2 catalyzes conjugation of ubiquitin moieties to SGK, leading to reduced levels of SGK [23].

There has been debate in the literature over detection of an interaction between SGK and Nedd4-2 *in vitro*, and the interaction is currently not understood at the molecular level. SGK contains a PY motif (PPFY), which might mediate interaction with the WW-domains of Nedd4/Nedd4-2. *In vitro* studies showed that Nedd4 and Nedd4-2 interact with wildtype SGK but not with SGK_Y298A_ that contains a mutated PY motif [19]. Two previous *in vitro* binding studies have asked whether the WW-domains of Nedd4-2 interact with an SGK peptide containing the PY motif. One study used surface plasmon resonance and concluded that interaction did occur [16], whereas the other study used intrinsic tryptophan fluorescence and did not observe an interaction [18]. Further, Rauh *et al*. [24] reported the lack of interaction between SGK and Nedd4-2 in a far-Western analysis. Here we report that SGK interacts with the WW-domains of Nedd4-2 and show that this interaction is functionally significant.

## Results

### SGK binds to WW-domains 2 and 3 of Nedd4-2

**Figure 1:**
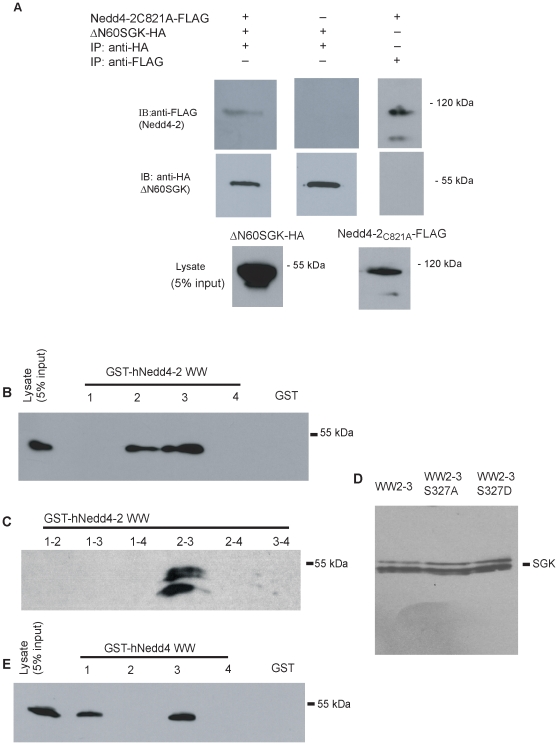
Nedd4-2 and Nedd4 WW-domains interact with SGK *in vitro*. (A) ΔN60SGK-HA and Nedd4-2_C821A_–FLAG were transiently co-expressed, or expressed alone as indicated, in COS7 cells. Complexes were immunoprecipitated with anti-HA antibody. Immunoprecipitates were analyzed by 10% SDS-PAGE and sequential western blotting (IB) with anti-FLAG antibody (Nedd4-2) and anti-HA antibody (ΔN60SGK). Expression of the proteins (5% input) is shown in the bottom panel, n = 3. Either ΔN60SGK-HA (B, E) or SGK-FLAG (C, D) was transiently expressed in COS7 cells. Cells were lysed 48 h after transfection with TBS +1% Triton-X-100. The lane labelled “lysate” shows expression of SGK in whole cell lysate. COS7 cell lysates were incubated with the indicated Nedd4-2 or Nedd4 WW-domain GST-fusion proteins, or GST alone, to test for interaction with SGK. Bound SGK was analyzed by immunostaining with anti-FLAG/HA antibody. The bands indicate that WW-domains 2 and 3 of Nedd4-2, and WW-domains 1 and 3 of Nedd4 interact with SGK, n = 7. The two SGK bands observed in some experiments are likely to be unphosphorylated and phosphorylated forms of SGK [8].

Co-immunoprecipitation was used to characterize an *in vitro* interaction between Nedd4-2 and SGK. Nedd4-2-FLAG and SGK-HA were co-expressed in COS7 cells, SGK was immunoprecipitated with anti-HA, and the presence of Nedd4-2 in the immunoprecipitates was assessed by western blotting with anti-FLAG. Nedd4-2 co-precipitated with SGK but the amount of Nedd4-2 co-precipitated was small, and appeared to be close to the limit of detection (data not shown). We reasoned that SGK would be rapidly turned over in the cells, and this would be enhanced by overexpression of active Nedd4-2, since Nedd4-2 is known to induce degradation of SGK [23]. Therefore we co-expressed a stable form of SGK (ΔN60SGK-HA, lacking the first 60 amino acids of SGK), together with a ligase-dead form of Nedd4-2 (Nedd4-2_C821A_–FLAG). After immunoprecipitating SGK and Western blotting for Nedd4-2 we show (Fig. 1A, top left panel) that SGK and Nedd4-2 interact when co-expressed in COS7 cells.

In order to better understand the molecular nature of the interaction between Nedd4-2 and SGK, *in vitro* GST pulldown studies were performed. The presence of a PY-motif in the SGK protein, a sequence that can be bound effectively by WW-domains in other contexts, suggests that the WW-domains of Nedd4-2 may be involved in mediating the interaction. Individual WW-domains of Nedd4-2 or Nedd4, or combinations of Nedd4-2 WW-domains, were expressed as GST fusion proteins and purified on glutathione-Sepharose beads. Lysates of COS7 cells expressing SGK or ΔN60-SGK (both SGK constructs gave the same results) were incubated with the WW-domain fusion proteins or GST alone. Bound SGK was detected by western blotting with anti-FLAG/HA. As shown in Fig. 1B, WW-domains 2 and 3 of Nedd4-2 individually bind SGK, whereas WW-domains 1 and 4 did not bind. Consistent with this finding, a GST fusion protein consisting of WW-domains 2 and 3 together with the intervening sequence also bound SGK (Fig. 1C). However, WW-domains 2 and 3 in other contexts did not readily bind to SGK (Fig. 1C). This suggests that additional sequences mask the WW-domains 2 and 3 binding sites in pulldown assays or that the folding of the larger constructs was incorrect.

There are three consensus SGK phosphorylation sites in Nedd4-2 and one of these, Ser327, lies between WW-domains 2 and 3. Therefore we tested whether mutation of Ser327 to alanine (to abolish any phosphorylation) or to aspartic acid (to mimic phosphorylation) would alter binding between SGK and Nedd4-2-WW2-3. GST pulldown assays (Fig. 1D) showed no differences in the binding of WW2-3, WW2-3_S327D_ or WW2-3_S327A_ to SGK.

The data from these GST interaction experiments served as a guide for subsequent surface plasmon resonance and molecular modelling experiments.

### SGK binds to WW-domains 1 and 3 of Nedd4

Although not a target for phosphorylation by SGK, Nedd4 has also been shown to interact with SGK [19]. The four WW-domains of Nedd4 were therefore also tested for their interaction with SGK using GST interaction assays (see Fig. 1E). In a similar manner to Nedd4-2, only two of the four WW-domains bound SGK. However, WW-domains 1 and 3 of Nedd4 interact with SGK, whereas for Nedd4-2 WW-domain 2 and 3 mediate binding to SGK. The profile of the SGK protein bound by Nedd4 and Nedd4-2 appeared to differ (compare Fig. 1 C and E). Other studies (e.g. [8]) report that different isoforms of SGK may be detected and these may reflect different phosphorylation states of SGK.

### SGK-PY-motif peptide binds to individual Nedd4-2 WW-domains WW2 and WW3 and most strongly to WW2–3

Surface plasmon resonance (SPR) was used to quantitate the findings from the GST pull-down assays, and to investigate the role of the PY-motif of SGK in this interaction. An SGK-PY-motif peptide was immobilized to the surface of the sensor chip using standard amine chemistry. GST fusion proteins containing the Nedd4-2 WW-domains, and WW-domain 3 of Nedd4, were cleaved with specific proteases to remove the GST moiety, and the isolated WW-domains purified to homogeneity by size exclusion chromatography. The pure WW-domains at a range of concentrations were then injected over the bound SGK-PY-motif peptide.

**Figure 2:**
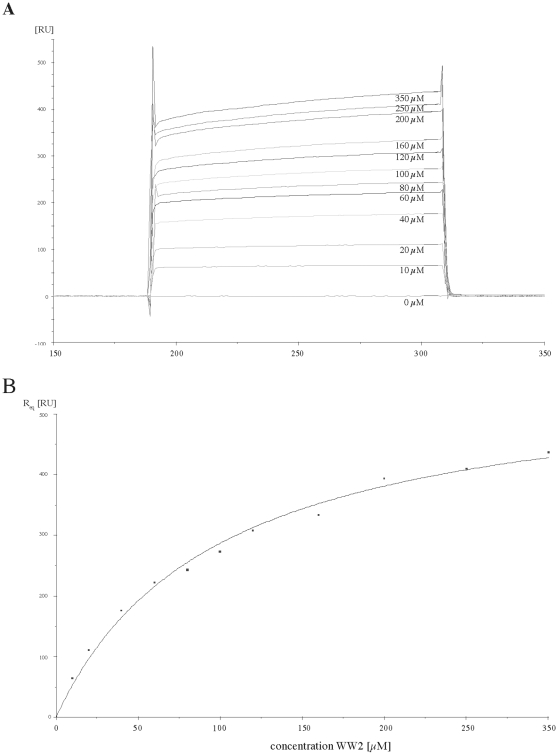
WW-domains 2 and 3 bind with low affinity to an SGK PY-motif peptide. (A) Typical BIAcore response curves produced by injecting different concentrations of the isolated Nedd4-2 WW2 domain over immobilized SGK PY-motif peptide. Each concentration was measured in duplicate. (B) Estimation of *K*_D_ from steady state equilibrium binding measurements. The equilibrium SPR response, *R*_eq_ was measured by taking the average value in the equilibrium region of the response curve between 120 and 180 seconds. *R*_eq_, was measured in triplicate for each concentration of WW2, and the mean value of *R*_eq_ was then plotted against the concentration of WW2. The solid line represents the best fit to the data using the equation described in Materials and Methods (χ^2^ = 0.34). Binding affinities of the other WW-domains are summarized in Table 1.

Fig. 2A shows a typical set of the resultant SGK-WW-domain binding curves. As shown previously for binding to ENaC, the WW-domains show very fast on- and off-rates, indicated by a “square wave” binding curve, which is typical for proteins with a low binding affinity. The equilibrium SPR responses were measured and plotted against the corresponding concentration of each WW-domain. A curve was fitted to the data that allowed the estimation of the *K*_D_ as the concentration of WW-domains required for half-maximal binding (Fig. 2B). This equilibrium binding approach was used previously in the analysis of SPR data of the interaction of the Nedd4 WW-domains with ENaC [15].

The calculated *K*_D_ values for the interaction of the WW-domains of Nedd4 and Nedd4-2 with the SGK PY-motif peptide are shown in Table 1. RNase was used as a negative control and showed no binding to the SGK PY-motif peptide. The interaction between the WW-domains 2 and 3 of Nedd4-2, and the SGK PY-motif peptide were in the micromolar range, with calculated *K*_D_ values of ∼100 µM and ∼250 µM respectively. Interestingly, the protein containing both of these WW-domains bound to the peptide significantly more strongly than either single WW-domain alone, with a *K*_D_ of ∼50 µM. In agreement with the GST pull-down assays, the WW-domains 1 and 4 of Nedd4-2 did not interact with the SGK-PY-motif peptide. The WW-domain 3 of Nedd4 was also tested for binding to the SGK peptide, and the affinity was determined to be similar to that of Nedd4-2 WW-domain 2, with a calculated *K*_D_ of ∼130 µM. Compared to the interaction between ENaC and the WW-domains of Nedd4 which was reported previously [15], [18], [25], the interaction between Nedd4/Nedd4-2 WW-domains and the SGK PY motif is 5 to 10 fold weaker.

**Table 1:** Binding affinities of the WW-domains of Nedd4 and Nedd4-2 to PY motif peptides from SGK and βENaC.

WW-domain	PY motif peptide	*K*_D_ (µM)
Nedd4-2 WW1	SGK	>600
Nedd4-2 WW2	SGK	105.0±9.9
Nedd4-2 WW3	SGK	249.5±7.8
Nedd4-2 WW4	SGK	>600
Nedd4-2 WW2-3	SGK	53.3±2.4
Nedd4 WW3	SGK	129.5±38.9
Nedd4-2 WW3	βENaC	11.1±0.4
RNase	SGK	>600

Values are mean ± S.E, n = 3–4.

### Molecular modelling analysis of the SGK-WW-domains interaction

Molecular modelling was undertaken to ascertain whether there were particular energetic factors that explained the preferential association between SGK and the Nedd4-2 WW-domains 2 and 3. In order to do this, the WW-domains and the SGK peptide were modelled in isolation, and as SGK-WW-domain complexes. The calculated relative binding energies predict that WW2 and WW3 bind to the SGK peptide more strongly than WW4 by approximately 70 and 60 kJ mol^−1^ respectively. Thus, as with the SPR-derived binding results, WW2 was shown to bind with highest affinity, closely followed by WW3. However, modelling was not able to predict the experimentally observed lack of affinity of the WW1 domain for the SGK peptide, which was calculated to have a similar affinity for the SGK peptide as WW3. Overall, there appears to be no single energy factor governing the strength of the SGK-WW-domain interaction, but rather a complex interplay between them.

### Activated SGK binds the WW-domains more strongly than inactivated SGK

**Figure 3:**
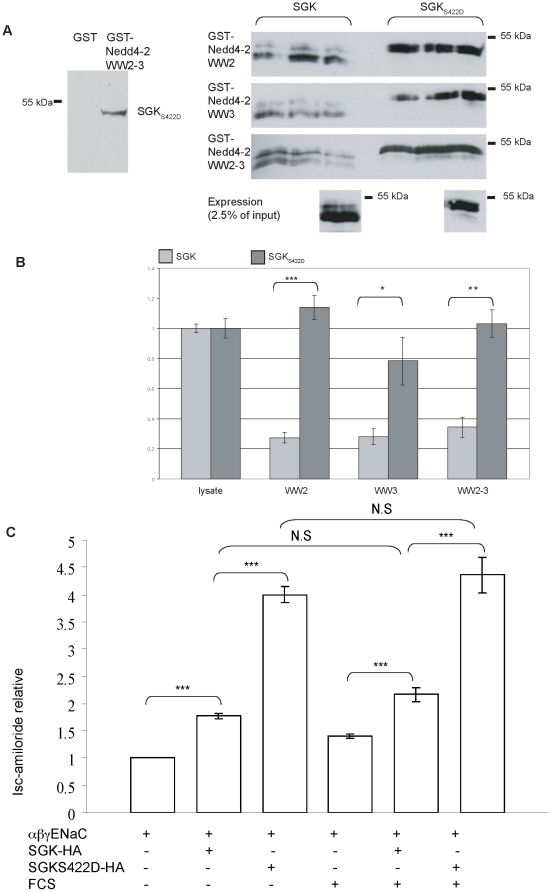
SGK_S422D_ binding to Nedd4-2 and stimulation of ENaC is enhanced compared to wildtype SGK. (A) SGK-FLAG or SGK_S422D_-FLAG were expressed in COS7 cells. After 48 hr cells were lysed, pre-cleared with GST bound to glutathione-Sepharose beads and then incubated with 50 µg of GST fusion proteins WW-domain 2, WW-domain 3, WW-domains 2 and 3 of Nedd4-2, or GST alone (left) as indicated. After washing the beads, the bound proteins were eluted and analyzed by SDS-PAGE and western blotting with anti-FLAG. The right panel shows interaction of SGK with the WW-domains, and also the interaction of SGK_S422D_ with the WW-domains. (B) The resulting bands were quantified by densitometry and normalized to total expression of either SGK or SGK_S422D_. Bars represent mean +/− S.E. (n = 9 for WW2 and WW3, n = 6 for WW2–3). (C) Relative amiloride-sensitive short-circuit sodium current in FRT cells after transfection with α-, β- and γENaC together with SGK-HA or SGK_S422D_-HA under serum (FCS) or serum-free conditions, n = 4. ***indicates *P*<0.001, **indicates *P*<0.01 and *indicates *P*<0.05, analysis of variance.

SGK is activated by phosphorylation, via a phosphatidylinositide 3-kinase (PI3K)-dependent pathway [26], and SGK is then able to phosphorylate substrates such as Nedd4-2. Although we were able to detect interaction of Nedd4-2 WW-domains with wildtype SGK (Fig. 1B, C), we hypothesized that an activated form of SGK might have a higher affinity for Nedd4-2. Therefore GST interaction experiments were performed comparing the binding of wildtype SGK and activated SGK (SGK_S422D_) to isolated Nedd4-2 WW-domains. SGK_S422D_ appeared to run more slowly compared to SGK, consistent with it representing a phosphorylated form of SGK [8]. SGK_S422D_ did not bind to GST alone (Fig. 3 A, left), and in parallel experiments we showed that SGK_S422D_ binds Nedd4-2 WW-domains more strongly than SGK (Fig. 3 A, B). This result indicates that phosphorylation may promote binding of SGK to Nedd4-2. To ask if SGK_S422D_ has an altered effect on Na^+^ transport, we measured the amiloride-sensitive short circuit current in Fischer rat thyroid (FRT) epithelia [27] transfected with α-, β-, and γENaC and either wildtype or activated SGK (Fig. 3C). SGK stimulated I_sc_-amiloride significantly, consistent with reports of others [6], [9]. SGK_S422D_ had a greater stimulatory effect on I_sc_-amiloride compared to SGK. Similar results were observed in both serum-containing and serum-free media, suggesting that lack of activation of wildtype SGK is not the primary reason for SGK_S422D_ being a more effective positive regulator of ENaC. Instead, increased binding of SGK_S422D_ to Nedd4-2 may be a factor in the improved ability of SGK_S422D_ to increase amiloride-sensitive current.

### SGK lacking the PY motif does not alter the Nedd4-2-ENaC inhibition dose response curve

**Figure 4:**
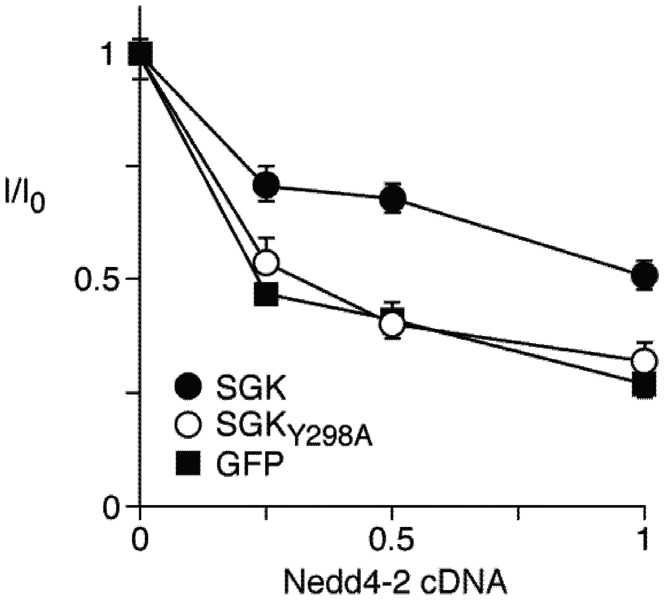
The SGK PY motif is necessary for SGK to inhibit Nedd4-2. Amiloride-sensitive short-circuit sodium current (relative to 0 µg of Nedd4-2) in FRT cells 48 hr after transfection with α-, β- and γENaC and SGK (closed circles) or SGK_Y298A_ (open circles) or GFP (closed squares), in the presence of increasing amounts of Nedd4-2, n = 18. Nedd4-2 is expressed as a fraction of the amount of ENaC cDNA. Expression of SGK and SGK_Y298A_ was reported in Figure 1B of Snyder *et al*. [19].

Our previous work has shown that SGK lacking its PY motif (SGK_Y298A_) is not able to bind full length Nedd4-2 (Fig. 1 in [19]), and others have shown that SGK lacking its PY motif is less able to phosphorylate Nedd4-2 [20]. To test the hypothesis that the interaction of the Nedd4-2 WW-domains with the SGK PY motif is required for SGK to influence Nedd4-2 function, we co-expressed α-, β- and γENaC in FRT epithelia with either wild type SGK or SGK_Y298A_ along with increasing amounts of Nedd4-2, and measured the amiloride-sensitive short circuit current. Consistent with our previous work, we found that in the absence of SGK, Nedd4-2 inhibited ENaC in a dose-dependent manner (Fig. 4) [11]. Wild type SGK reduced inhibition, shifting the dose-response relationship to the right (Fig. 4). In contrast, SGK_Y298A_, that has a disrupted PY motif, had no effect on the Nedd4-2 dose-response relationship (Fig. 4). A previous report has shown that SGK with a mutated PY motif tyrosine retains catalytic activity similar to that of wildtype SGK [20]. Therefore, the requirement for the PY motif suggests that in order for SGK to inhibit Nedd4-2, it must bind to one or more of the Nedd4-2 WW-domains.

## Discussion

In this study we confirmed that the WW-domains of Nedd4 and Nedd4-2 mediate binding to SGK. The binding appears to be relatively weak, but is enhanced when SGK is activated, and the activated form of SGK induces higher levels of Na^+^ transport in ENaC-transfected epithelia. We also used a functional assay to show that the PY motif of SGK is essential for preventing Nedd4-2 inhibition of ENaC by SGK.

Using pulldown assays and SPR we showed that the WW-domains 2 and 3 of Nedd4-2 both bind individually to SGK, but that a protein construct containing both of these WW-domains and the intervening sequence binds more strongly to SGK than either domain on its own, with a dissociation constant of 53 µM. This binding is ∼5 fold weaker than previously reported results for Nedd4/Nedd4-2 WW-domain binding to ENaC PY motifs [15], [16], [18]. Our data may be an overestimate of the binding affinity due to the difficulty of purifying sufficient soluble WW-domain proteins at a concentration greater than twice the calculated *K*_D_. This suggests that the binding of Nedd4-2 to SGK is rather weak, and hence possibly transient *in vivo*.

Our data are consistent with that reported by Asher *et al*. [16] who found that the binding of Nedd4-2 WW-domains to SGK was much weaker than their binding to ENaC, although no affinity constants were reported. Asher *et al*. [16] reported that Nedd4-2 WW-domain 3 bound best to the SGK PY motif peptide followed by tandem WW-domain proteins: WW-domains 2 and 3 and WW-domains 3 and 4. Our results show that the tandem protein containing WW-domains 2 and 3 binds best and that WW domain 2 binds more strongly than WW domain 3. The studies of Asher *et al*. used fusion proteins with the GST moiety intact whereas in our studies the GST moiety, which can dimerize and hence affect the results, was removed. These data differ to that of Henry *et al*. [18] and Rauh *et al*. [24] who did not detect binding of SGK to Nedd4-2 WW-domains. Rauh *et al*. have suggested that species difference may account for these discrepancies as *in vitro* interactions have been reported for the human, but not the mouse or rat proteins [24]. Our data, using human SGK and human Nedd4-2 support this hypothesis, although the SGK PY motif peptides used in the studies were slightly different, and the use of different techniques may also contribute to the differences. Further, as shown in Figure 1, we report a robust *in vitro* interaction between a stable form of SGK and ligase-dead Nedd4-2, suggesting that the difficulties in detecting interaction between the full-length forms of SGK and Nedd4-2 proteins are due to rapid degradation of SGK that is enhanced in the presence of wild-type Nedd4-2.

The multiple WW-domain 2 and 3 construct from Nedd4-2 (WW2-3) used in the present study shows a higher binding affinity for the SGK PY motif peptide (*K*_D_ = 53 µM) than either WW-domain in isolation (*K*_D_ = 105 or 249 µM). This is in marked contrast to the behaviour of a multiple WW-domain construct (WW domains 2, 3, and 4) from Nedd4 binding to the βENaC PY motif peptide less strongly (*K*_D_ = 65 µM) than WW3 alone (*K*_D_ = 11.1 µM) as we reported previously [15]. The fact that Nedd4-2 WW2-3 shows increased affinity for the SGK PY motif peptide compared to its constituent domains implies that a cooperative interaction is occurring between the WW-domains, that is not seen in the binding of the Nedd4 multiple WW-domain construct to the βENaC PY motif peptide. Therefore, there is presumably some contribution to this cooperative binding from the intervening sequence. This might arise either by the binding state of one WW-domain being able to influence the affinity of the other for its target, or through possible different orientations of the two WW-domains in the Nedd4-2 WW2-3 protein compared to its Nedd4 counterpart. This region of the polypeptide is the most different in sequence between Nedd4 and Nedd4-2, with significant insertions in Nedd4-2: 39 extra residues between WW domain 2 and WW domain 3. We tested whether presence of an SGK phosphorylation site between the domains might influence binding, however changing Ser327 to either Ala or Asp did not alter binding to SGK (Fig. 1D).

The presence of cooperative binding in Nedd4-2, but not in Nedd4 supports other data showing that the structural context of the WW-domains is able to influence their affinity for peptide ligands, making it possible that the true *in vivo* binding affinity between SGK and Nedd4-2 may be tighter than that measured in our *in vitro* system. Jennings *et al*. [28] have reported that tandem arrangement of WW-domains in *suppressor of deltex* influences ligand binding, such that binding of ligand to one WW-domain enhances ligand binding to a nearby WW-domain.

The finding, in GST pulldown assays (Fig. 1C), that WW2–3 cannot mediate binding to SGK when other WW-domains are present may be an artifact due to a sequence in the longer multiple WW-domain constructs being unable to fold correctly or due to occlusion of the binding site in this context preventing WW-domains 2 and 3 from binding to SGK. However, the results are consistent with the hypothesis that intervening sequences influence binding of Nedd4-2 WW-domains to SGK.

SGK must be phosphorylated on two residues before it is an active kinase, and mutation of one of these sites (SGK_S422D_) is sufficient to produce active SGK protein [29]. Here we show that SGK_S422D_ binds more strongly to Nedd4-2 WW-domains compared to wildtype SGK, and that SGK_S422D_ is more effective in stimulating I_sc_-amiloride in ENaC-transfected epithelia. Lack of activation of SGK is unlikely to be the cause of the difference in stimulation of current as the effect was similar in epithelia incubated in both serum-containing and serum-free media. However, we cannot exclude the possibility that kinase activity has changed in SGK_S422D_. These results contrast with studies on other channels and transporters in *Xenopus* oocytes that reported similar activation effects of SGK and SGK_S422D_ on the Na^+^-coupled glucose transporter SGLT1 [30] and the voltage-gated sodium channel SCN5A [31]. This suggests that SGK activation of ENaC may be controlled differently to SGLT1 and SCN5A, or that the SGK signalling pathway differs between *Xenopus* oocytes and FRT cells.

Finally, our functional data show the importance of the PY motif of SGK in mediating its ability to prevent Nedd4-2 inhibition of ENaC, extending previous reports [20].

Together our data support the model that activated SGK binds to WW-domains 2 and 3 of Nedd4-2, then phosphorylates Nedd4-2 on one or more sites. This produces a binding site for 14-3-3 family members to bind Nedd4-2 and sequester it away from ENaC. The result is an increase in ENaC at the cell surface, and activation of amiloride-sensitive sodium current.

## Materials and Methods

### cDNA constructs

Nedd4-2-FLAG, SGK_S422D_-FLAG, SGK-HA (hemagglutinin), SGK_S422D_-HA, ΔN60SGK-HA, ΔN60SGK_S422D_-HA (lacking the first 60 amino acids of SGK) all containing the indicated epitope tags at the C-terminus were cloned into pMT3. Nedd4-2_C821A_–FLAG [23], Nedd4_2S327A/D_ [7], FLAG-SGK and FLAG-SGK_Y298A_ [19] have been described previously. GST fusion proteins containing individual WW-domains of Nedd4-2 and Nedd4 are described elsewhere [11], [32]. Nedd4-2/Nedd4LA GST fusions contained the following amino acids: WW1 (PPP….SSE), WW2 (TTP….EDG), WW3 (TQS…VHM), WW4 (DLG….AIT). Primer pairs for production of Nedd4 WW-domains are documented in [32]. To prepare GST fusion proteins containing multiple WW-domains of Nedd4-2, pairs of primers surrounding the appropriate WW-domains were used. DNA was sequenced at the Allan Wilson Centre Genome sequencing service, Massey University, Palmerston North, New Zealand.

### Cell culture and transient transfection

COS7 cells [11] were grown in Dulbecco's modified Eagle's medium (DMEM) containing 1.5 g/L sodium bicarbonate, supplemented with 10% fetal calf serum, 10 units/ml penicillin, and 10 mg/ml streptomycin. Cells were maintained at 37°C and 5% CO_2_. The day before transfection, COS7 cells were plated at a density of 3×10^5^ cells in 35 mm plates. COS7 cells were transfected with 1.5 µg of each cDNA construct using FuGENE 6 (Roche Diagnostics, Mannheim, Germany) as described [32].

### Co-immunoprecipitation

COS7 cells were co-transfected with ΔN60SGK-HA and Nedd4-2_C821A_-FLAG. Cells were lysed 48 h after transfection in T-TBS (150 mM NaCl, 50 mM Tris, pH 7.4+1% Triton-X-100 (BioRad, Hercules, CA, U.S.A.)), the lysate was incubated with 25 µg/ml anti-HA antibody (Sigma, St Louis, MO, U.S.A.) for 2 h at 4°C. Then 25 µl of protein G-Sepharose beads (Sigma) was added and incubation continued for 1 h at 4°C. The beads were washed four times in T-TBS and analysed by SDS-PAGE and Western blotting with anti-FLAG (Sigma).

### GST binding experiments

pGEX-KG containing the cDNA of WW-domains of Nedd4 and Nedd4-2 or the empty vector were transformed into *Escherichia coli* strain BL21, and expression of the WW-domains-GST fusion protein or GST alone was induced with 0.5 µM isopropyl-D-1-thiogalactopyranoside for 3 h at 37°C. Bacteria were lysed using BugBuster (Novagen, Merck KGaA, Darmstadt, Germany), and the GST fusion proteins were purified on glutathione-Sepharose beads (GE Healthcare, Little Chalfont, Bucks, U.K.). COS7 cells were transiently transfected with pMT3 containing SGK/SGK_S422D_-FLAG or ΔN60SGK-HA. After 48 h expression cells were lysed in T-TBS. Insoluble material was pelleted by centrifugation (5 min, 16,000×g), and the lysates were precleared for 1 h at 4°C with GST bound to glutathione-Sepharose beads. The precleared lysates were then incubated with WW-domain GST fusion proteins or GST alone for 3 h at 4°C. The glutathione-Sepharose beads, along with the fusion proteins and any binding proteins, were collected by centrifugation (1 min, 16,000 *g*), washed extensively in T-TBS and analyzed by SDS-PAGE and Western blotting using anti-FLAG antibody. Resulting X-ray films were analyzed densitometrically.

### Measurement of WW-domain binding to SGK peptide using SPR

GST-WW-domain fusion proteins were prepared in bulk, as described above, except that the bacterial pellet was resuspended in HBS buffer (10 mM HEPES, 150 mM NaCl, 3 mM EDTA, pH 8.0) supplemented with protease inhibitors. After cell lysis by sonication, Triton X-100 was added to a final concentration of 1%, the insoluble material was removed by centrifugation and glutathione-Sepharose beads were added to the supernatant. After a 2 hr incubation at 4°C the beads were collected by centrifugation, washed and resuspended in HBS. To release the WW-domains from the GST moiety, 150 units of thrombin protease or recombinant tobacco etch virus protease and 950 µl of PBS were added to 5.0–7.5 mg of fusion protein. Cleavage took place at room temperature for 4 hr. The proteins were finally purified to apparent homogeneity (as judged by Coomassie stained SDS-PAGE), using a Superdex-75 16/60 column (GE Healthcare), and concentrated using a Millipore ultrafiltration unit. Protein concentration was measured using absorbance at 280 nm [33].

An SGK peptide containing the PY motif was synthesized: NH_2_-CLYGLPPFYSRNT- CONH_2_ and for comparison a βhENaC PY-motif peptide was used, NH_2_-GTPPPNYDSLR-CONH_2_ (Auspep, Parkville, Australia). For SPR experiments using a BIAcore 2000 system (BIAcore AB, Uppsala, Sweden) [34] the SGK and β peptides were attached either via the N-terminal amino group or via a C-terminal arginine side chain to a carboxylated surface (Sensor Chip CM5) using standard amine immobilisation chemistry. Briefly, the carboxy surface was activated with 1-(3-diethylaminopropyl)-3-ethylcarbodiimide and *N*-hydroxysuccinimide. Peptides were dissolved in 100 mM Na-borate buffer, pH 8.6 to give a final concentration of 300–1000 µg/ml. This solution was injected over the activated carboxylated surface at a flow rate of 20 µl/min, and unreacted surface esters were subsequently blocked with 1 M ethanolamine, pH 8.5. The amount of peptide immobilized was controlled by manually varying the contact time. Interaction studies were carried out by injecting WW-domain-containing proteins over this surface at 20 µl/min, using HBS-EP as running buffer. The signal from a blank flowcell, (which had been activated and blocked as above, but to which no peptide had been immobilized) was subtracted from all SPR measurements to correct for bulk solvent effects. Data were analyzed using the Biacore BIAevaluation software (v3.1). The affinity constant, *K*_D_, was estimated by evaluation of steady state affinity data from a series of different concentrations of WW-domains, using the formula *R*_eq_ = *CR*_max_/(*Cn*+1/*K*_D_), where *R*_eq_ is the steady state binding response, *C* is the protein concentration, *R*_max_ is the theoretical maximum binding capacity of the surface, and *n* is the steric interference factor, which specifies how many binding sites are on average blocked by the binding of one analyte molecule, which was assumed to be one.

### Molecular modelling of WW-domains bound to SGK peptide

Modelling studies were performed following the protocol reported in Lott *et al*. [15]. Briefly, Nedd4-2 WW-domains alone, the SGK peptide and their respective complexes were independently modelled using the Amber94 forcefield, using cycles of molecular dynamics and minimization to determine global minimum energy structures. Binding energies for the complexes formed between each WW-domain and the SGK peptide were determined relative to the isolated components.

### Electrophysiology

Fischer rat thyroid (FRT) cells [27] were grown on permeable filter supports (Snapwell™, Corning USA or Millicell PCF, both with 0.4 mm pore size, 12 mm diameter) in F-12 Coon's media with 5% fetal calf serum (Sigma), 100 U/ml penicillin, and 100 µg/ml streptomycin at 37°C. One day after seeding, cells were cotransfected with α-, β- and γENaC (0.07 µg each), 0.6 µg wildtype or mutant SGK, and 0–0.2 µg Nedd4-2 (Fig. 4 only). Total DNA was held constant (1 µg/millicell) using cloning vector DNA. The plasmids were mixed with Lipofectamine 2000 (Invitrogen) or TFX 50 (Promega, Madison, WI; 7.9 µg/millicell) in serum free F12 Coon's media for 15 minutes and transferred to the apical surface of the monolayer. One-six hours later, the apical media was replaced with F-12 Coon's media containing 5% fetal calf serum and amiloride (10 µM). Short-circuit current was measured 3 days after transfection in modified Ussing chambers connected to a voltage clamp (South Campus Electronics, Dunedin, New Zealand or Warner Instruments). The apical and basolateral surfaces were bathed in (in mM) 135 NaCl, 1.2 CaCl_2_, 1.2 MgCl_2_, 2.4 K_2_HPO_4_, 0.6 KH_2_PO_4_, 10 HEPES, pH 7.4, at 37°C and bubbled with O_2_. Amiloride-sensitive short-circuit current (I_sc_-amiloride) was determined as the difference in current with and without amiloride (10 µM) in the apical bathing solution. Data are presented as relative I_sc_-amiloride (I_sc_-amiloride relative) that was obtained by normalizing the I_sc_-amiloride to that of control cells transfected with αβγENaC in the same experiment. Transepithelial resistance (R_t_) was monitored by applying repetitive voltage potential pulses (5 mV for 1 sec at 120 sec intervals) across the epithelium.

## References

[1] Garty H, Palmer LG (1997). Epithelial sodium channels: function, structure, and regulation.. Physiol Rev.

[2] Snyder PM, Steines JC, Olson DR (2004). Relative contribution of Nedd4 and Nedd4-2 to ENaC regulation in epithelia determined by RNA interference.. J Biol Chem.

[3] Snyder PM (2005). Minireview: regulation of epithelial Na+ channel trafficking.. Endocrinology.

[4] Hughey RP, Bruns JB, Kinlough CL, Harkleroad KL, Tong Q (2004). Epithelial sodium channels are activated by furin-dependent proteolysis.. J Biol Chem.

[5] Bruns JB, Carattino MD, Sheng S, Maarouf AB, Weisz OA (2007). Epithelial Na+ channels are fully activated by furin- and prostasin-dependent release of an inhibitory peptide from the gamma-subunit.. J Biol Chem.

[6] Chen SY, Bhargava A, Mastroberardino L, Meijer OC, Wang J (1999). Epithelial sodium channel regulated by aldosterone-induced protein sgk.. Proc Natl Acad Sci U S A.

[7] Snyder PM, Olson DR, Kabra R, Zhou R, Steines JC (2004). cAMP and serum and glucocorticoid-inducible kinase (SGK) regulate the epithelial Na(+) channel through convergent phosphorylation of Nedd4-2.. J Biol Chem.

[8] Wang J, Barbry P, Maiyar AC, Rozansky DJ, Bhargava A (2001). SGK integrates insulin and mineralocorticoid regulation of epithelial sodium transport.. Am J Physiol Renal Physiol.

[9] Náray-Fejes-Tóth A, Canessa C, Cleaveland ES, Aldrich G, Fejes-Tóth G (1999). sgk is an aldosterone-induced kinase in the renal collecting duct.. J Biol Chem.

[10] Kamynina E, Tauxe C, Staub O (2001). Distinct characteristics of two human Nedd4 proteins with respect to epithelial Na+ channel regulation.. Am J Physiol Renal Physiol.

[11] McDonald FJ, Western AH, McNeil JD, Thomas BC, Olson DR (2002). Ubiquitin-protein ligase WWP2 binds to and downregulates the epithelial Na(+) channel.. Am J Physiol Renal Physiol.

[12] Goulet CC, Volk KA, Adams CM, Prince LS, Stokes JB (1998). Inhibition of the epithelial Na^+^ channel by interaction of Nedd4 with a PY motif deleted in Liddle's syndrome.. J Biol Chem.

[13] Shi PP, Cao XR, Sweezer EM, Kinney TS, Williams NR (2008). Salt-sensitive hypertension and cardiac hypertrophy in mice deficient in the ubiquitin ligase Nedd4-2.. Am J Physiol Renal Physiol.

[14] Snyder PM, Olson DR, McDonald FJ, Bucher DB (2001). Multiple WW domains, but not the C2 domain, are required for inhibition of the epithelial Na+ channel by human Nedd4.. J Biol Chem.

[15] Lott JS, Coddington-Lawson SJ, Teesdale-Spittle PH, McDonald FJ (2002). A single WW domain is the predominant mediator of the interaction between the human ubiquitin-protein ligase Nedd4 and the human epithelial sodium channel.. Biochem J.

[16] Asher C, Sinha I, Garty H (2003). Characterization of the interactions between Nedd4-2, ENaC, and sgk-1 using surface plasmon resonance.. Biochim Biophys Acta.

[17] Fotia AB, Dinudom A, Shearwin KE, Koch JP, Korbmacher C (2002). The role of individual Nedd4-2 (KIAA0439) WW domains in binding and regulating epithelial sodium channels.. Faseb J.

[18] Henry PC, Kanelis V, O'Brien MC, Kim B, Gautschi I (2003). Affinity and specificity of interactions between Nedd4 isoforms and the epithelial Na+ channel.. J Biol Chem.

[19] Snyder PM, Olson DR, Thomas BC (2002). Serum and glucocorticoid-regulated kinase modulates Nedd4-2-mediated inhibition of the epithelial Na+ channel.. J Biol Chem.

[20] Debonneville C, Flores S, Kamynina E, Plant PJ, Tauxe C (2001). Phosphorylation of Nedd4-2 by Sgk1 regulates epithelial Na+ channel cell surface expression.. EMBO J.

[21] Snyder PM (2009). Down-regulating destruction: phosphorylation regulates the E3 ubiquitin ligase Nedd4-2.. Sci Signal.

[22] Liang X, Peters KW, Butterworth MB, Frizzell RA (2006). 14-3-3 isoforms are induced by aldosterone and participate in its regulation of epithelial sodium channels.. J Biol Chem.

[23] Zhou R, Snyder PM (2005). Nedd4-2 phosphorylation induces serum and glucocorticoid-regulated kinase (SGK) ubiquitination and degradation.. J Biol Chem.

[24] Rauh R, Dinudom A, Fotia AB, Paulides M, Kumar S (2006). Stimulation of the epithelial sodium channel (ENaC) by the serum- and glucocorticoid-inducible kinase (Sgk) involves the PY motifs of the channel but is independent of sodium feedback inhibition.. Pflugers Arch.

[25] Asher C, Chigaev A, Garty H (2001). Characterization of interactions between Nedd4 and β and γENaC using surface plasmon resonance.. Biochem Biophys Res Comm.

[26] Kobayashi T, Cohen P (1999). Activation of serum- and glucocorticoid-regulated protein kinase by agonists that activate phosphatidylinositide 3-kinase is mediated by 3-phosphoinositide-dependent protein kinase-1 (PDK1) and PDK2.. Biochem J.

[27] Sheppard DN, Carson MR, Ostedgaard LS, Denning GM, Welsh MJ (1994). Expression of cystic fibrosis transmembrane conductance regulator in a model epithelium.. Am J Physiol Lung Cell Mol Physiol.

[28] Jennings MD, Blankley RT, Baron M, Golovanov AP, Avis JM (2007). Specificity and autoregulation of Notch binding by tandem WW domains in suppressor of Deltex.. J Biol Chem.

[29] Kobayashi T, Deak M, Morrice N, Cohen P (1999). Characterization of the structure and regulation of two novel isoforms of serum- and glucocorticoid-induced protein kinase.. Biochem J.

[30] Dieter M, Palmada M, Rajamanickam J, Aydin A, Busjahn A (2004). Regulation of glucose transporter SGLT1 by ubiquitin ligase Nedd4-2 and kinases SGK1, SGK3, and PKB.. Obes Res.

[31] Boehmer C, Wilhelm V, Palmada M, Wallisch S, Henke G (2003). Serum and glucocorticoid inducible kinases in the regulation of the cardiac sodium channel SCN5A.. Cardiovasc Res.

[32] Farr TJ, Coddington-Lawson SJ, Snyder PM, McDonald FJ (2000). Human Nedd4 interacts with the human epithelial Na^+^ channel: WW3 but not WW1 binds to Na^+^-channel subunits.. Biochem J.

[33] Gill S, Hippel P (1989). Calculation of protein extinction coefficients from amino acid sequence data.. Anal Biochem.

[34] Jönsson U, Fägerstam L, Ivarsson B, Johnsson B, Karlsson R (1991). Real-time biospecific interaction analysis using surface plasmon resonance and a sensor chip technology.. Biotechniques.

